# Aluminium Bronze in Medicine and Dentistry

**Published:** 1886-10

**Authors:** 


### SELECTIONS.

### Aluminium Bronze in Medicine and Dentistry.

Prof. C. Sauer, of Berlin, says in regard to this substance: Aluminium bronze, which I have utilized for the under layer of teeth-plates, is entirely free from injurious oxidation. For this purpose and in teeth-straightening machines, it admits of easier manipulation than gold alloys or silver. I believe, therefore, that it might be of use in the manufacture of surgical instruments. Canulas, sounds, catheters, etc., might be made, and everything that is manufactured from silver. It is an alloy of 900 copper and 100 aluminium. It admits of almost as ready stamping and pressing as pure silver (which next to pure gold is the softest metal), and it has besides the elasticity of steel. In form of wire, aluminium bronze possesses a power of resisting tension approaching that of steel wire. These characteristics render the aluminium bronze capable of substitution in many cases for silver, and for silver and gold alloys. I have found the melting-point of the aluminium bronze to be higher than that of pure gold, 1,000° C. It may, accordingly, be made red-hot without danger of melting, and manipulated with hard solder. I solder it with from 14 to 16-carat red gold, and such solder is more capable of resisting chemical influences than the silver solder, which contains zinc. 10 grms. of aluminium bronze, in the form of a lead plate, are worth 25 pfennige (6 cents), while 10 grms. of 12-carat silver, in the form of a lead plate, are worth 1 mark 50 pfennige (37 cents); 10 grms. 14-carat gold cost 19 marks ($4.75). Aluminium bronze is one-half lighter than 12-carat silver, and almost half the weight of 14-carat gold.

For testing how aluminium bronze is preserved in the mouth, I have employed the galvanic current. Pure aluminium, with other metals, in the presence of an acid or an alkali, yields a galvanic current. Years ago, in experiments with the mouth, I found that in a combination of aluminium with zinc the latter oxidizes. In manufacturing a combination of aluminium with gold alloy or platinum, under such circumstances, the aluminium, on the contrary, decomposes. In two cases in which it was possible for me to weigh pieces of aluminium bronze placed in the mouth, under the influence of a possible galvanic current, and the pieces not being cleaned, and therefore not mechanically worn, after the lapse of four weeks I noticed no loss of weight. For instance, with a piece weighing 16 grms., I have been able to discover only a loss of 0.008, and besides, after first weighing the piece, a screw was afterwards inserted, so a very slight mechanical loss of weight, therefore, took place. The aluminium bronze oxidizes only superficially in the mouth. There forms upon it a kind of patina, such as is formed in the weighing of plates of 14, 16, 18, and even 20-carat gold. It admits of manufacture into spiral springs, plates, screws, canulas, etc., for surgical purposes. Even knives have been manufactured therefrom. A solution of corrosive sublimate of 1 to 1,000 affects it superficially. For its disinfection carbolic acid is to be preferred, as it does not attack the aluminium bronze. Gold aluminium bronze acts similarly, but oxidizes to a greater extent, is softer, and not so elastic, and therefore is to be used as green gold or 20-carat gold is used.—*Therapeutic Gazette*, June 15, 1886.

